# The use of professional help and predictors of unmet needs for dealing with mental health to legal problems among victims of violence, accidents, theft and threat, and nonvictims in the general population

**DOI:** 10.1371/journal.pone.0259346

**Published:** 2021-11-17

**Authors:** Peter G. van der Velden, Carlo Contino, Pien van de Ven, Marcel Das

**Affiliations:** 1 CentERdata, Tilburg, The Netherlands; 2 Tilburg University’s Network on Health and Labor (NETHLAB), Tilburg, The Netherlands; 3 Fonds Slachtofferhulp, The Hague, The Netherlands; 4 Netherlands Institute for the Study of Crime and Law Enforcement, Amsterdam, The Netherlands; 5 Slachtofferhulp Nederland, Utrecht, The Netherlands; 6 Tilburg School of Economics and Management, Tilburg University, Tilburg, The Netherlands; Universidad Pública de Navarra, SPAIN

## Abstract

**Aims:**

Victims of violence, accidents, theft, and serious threat (hereafter abbreviated as victims) are more than nonvictims at risk for problems in different domains, varying from mental health to legal problems. However, the extent to which victims with these problems compared to nonvictims with similar problems receive problem-related professional or formal help is unclear. It is unknown if predictors of unmet needs differ between victims and nonvictims. Aim of the present study is to fill this gap of knowledge.

**Methods:**

Data was extracted from surveys of the VICTIMS-study (2018, 2019 and 2020), conducted with the Dutch population-based longitudinal LISS panel. Each survey assessed 1.) experiences with physical violence, accidents, theft, and serious threat and other traumatic or stressful events in the past 12 months and 2.) various problems and use of professional help, e.g. do receive help, do not need help, could use help but do not use it, cannot find or afford help for these problems. Multivariate logistic regression analyses were performed to assess differences in problems, in the use of problem-related professional help, and in predictors of unmet needs between victims (N = 1,756) and nonvictims (N = 5,000).

**Results:**

Victims more often had assessed problems than nonvictims. Victims compared to nonvictims with similar problems had 1.5 to 2 times more often unmet needs: they could not find or afford professional help for their mental, physical, partner/family, financial and legal problems. In addition, victims less often received help for legal and administrative problems. Most predictors of unmet needs, e.g. could use help but do not use it, cannot find or afford help, were not significant and hardly differed between both groups.

**Conclusions:**

The findings that victims compared to nonvictims more often have various problems and more often cannot find or afford problem-related professional help, suggest that there is room for improvement for victims services.

## Introduction

Adult victims of physical, sexual and partner violence, accidents, theft, and serious threat may face problems varying from mental health and work to financial and legal problems [1–6]. In addition, these problems may be interrelated. Sustained mental or physical injuries may result in compensation-related legal problems and sickness leave. Work- and financial problems may increase the risk for post-event mental health problems [7–10].

An important question for victims services and policymakers is to what extent victims with these problems have access to and use professional or formal help to reduce or solve these problems, such as mental health professionals, lawyers, physicians, and financial experts. If many victims with problems do not have access to or do not use professional help, victims services and policymakers should develop interventions and policies, or re-design existing interventions and policies, to target this gap [11].

To date, a large number of studies have been devoted to this question. Part of these studies is rooted in psychological and psychiatric research on post-trauma mental health problems, and part is rooted in criminological and socio-legal research on the effects of crime [12]. Both research domains focus on partly different aspects of services use and type of professional services complicating the comparison of outcomes. In addition, in both research domains relatively few studies focused on services use among victims of (traffic) accidents.

### Use of services

Psychological and psychiatric studies mainly focused on mental health services use or receiving treatment among victims with mental health problems and particularly PTSD. The main conclusion that can be drawn from these studies is that many victims with mental health problems such as PTSD do not (currently) use mental health services or receive treatment [12–14]. For instance, analyses of 26 population surveys in the World Health Organization’s World Mental Health Surveys showed that across countries and type of potentially traumatic events, about 50% of people with 12-month PTSD used some type of mental health treatment in the past 12 months [13]. Of the studies assessing services use after specific PTEs, most studies focused on victims of sexual assault and intimate partner violence [15–17]. These studies show that many victims of these events did not seek or use mental health services [12, 18–20].

In general, research in the criminological and socio-legal domain pays somewhat more attention to the use of different types of professional help. They also show that many victims do not use professional help, such as reporting the event to law enforcement and use of medical services. For instance, Cho et al. [11] analyzed the US National Crime Surveys data of 1992 to 2015 on the use of formal help for feelings victims experienced in the 6 months after experiencing the criminal event [21]. Results showed that 60.5% of the female victims of physical IPV used legal help, 23.6% used medical help, and 69.4% used medical and legal help in the 6 months before the assessment. However, in this study respondents reporting multiple victimization were excluded. Legal help in this study was defined as “were the police informed about this incident in any way”. As stated above, relatively few empirical studies on services use focused on victims of (traffic) accidents, while 50–60% of major trauma hospital admissions in Western countries are due to road traffic accidents [22–25]. The study by Ruseckaite et al. [23] for example, assessed visits to 19 different health care providers in the 12 months after hospitalization among traffic accidents victims with accepted claims in Australia between 1995 and 2008. Findings showed that 95.7% visited a medical professional other than a general practitioner, 34.7% a physiotherapist, 4.0% a counsellor or psychologist, 1.6% a speech therapist and 0.2% a psychiatrist during these 12 months.

### Predictors of unmet needs

For victims services and policy makers it is also relevant to know which subgroups of victims more often have unmet needs, e.g. could use help for their problems but do not use it or cannot find or afford problem-related help, compared to nonvictims with unmet needs for similar problems. The review of McCart et al. [12] on help seeking among victims of crime concluded that females, older adults, white adults, single and divorced adults were more likely to use mental health services. In addition, the review of Elhai et al. [26] on health services use (among the survivors of various traumatic events) concluded that mental health services use was generally related to being female, having a previous trauma history and having a PTSD diagnosis, and medical services use was related with PTSD diagnosis. With respect to the use of victims services, Lowe et al. [27] found that the number of contacts was not related to gender, ethnicity, type of crime, past minor or major offences, practical needs in contrast to intoxication (when victimized) status, risk level and psychological needs. In the study of Ford-Gilboe et al. [28], the use of social services was independently associated with low income and education, dependent children, and high chronic pain disability; the use of violence specific services with low income and ongoing abuse; and the use of legal services, dependent children and PTSD symptoms; and the use of mental health services with age, depressive symptoms and pain disability. In the study by Lipsky and Caetano [29], Hispanic and non-Hispanic white victims of IPV significantly more often than nonvictims reported that during the past 12 months there was any time when they needed mental health treatment or counseling but did not get it (while controlling for, among others, alcohol and illicit drug use, abuse and dependency).

### Research questions

The main finding across studies that many victims do not use professional help suggest that there is room for improvement in the use of professional help among victims with mental health, physical health, work, partner/ family, financial, administrative, and legal problems. However, several studies on services use do not specifically assess services use among victims who suffer from specific problems [13], which may underestimate services use. Having problems without receiving professional help is not equivalent to unmet needs: many victims are able to solve problems without professional help. In addition, a complicated ethical issue is beyond which level the prevalence of problem-related services use must be considered problematic and prove that access and services use must be improved. To the best of our knowledge there are no general accepted criteria for mental health, physical health, work, partner/ family, financial, administrative, and legal problems that guide decisions on this matter, other than that all victims with problems should have access to professional help particularly when unable to solve these problems.

An alternative to assist in solving this ethical issue is to compare prevalence of services use among victims with problems, with the prevalence of services use among nonvictims who are suffering from similar problems. When victims with problems compared to nonvictims with problems significantly less often use professional help, this may serve as an indication that services use must be improved. Forn-Gilboe et al. [28] compared the rates of services use of women who have separated from an abuse partner with population rates. They found that service use rates were substantially higher than population estimates for health, social, legal, and violence specific services, particularly for general and mental health services. In the study by Lipsky and Caetano [29], Hispanic and non-Hispanic white victims of IPV significantly *more* often than nonvictims reported that during the past 12 months there was any time when they needed mental health treatment or counseling but did not get it, while controlling for, among others, alcohol and illicit drug use, abuse and dependency. However, to the best of our knowledge, population-based studies that systematically assessed differences in the use of professional services for mental health, physical health, work, partner/ family, financial, administrative, and legal problems between victims and nonvictims with similar problems are absent.

In addition, we are not aware of empirical studies that compared predictors of unmet needs related to mental health to legal problems among victims with predictors of unmet needs related to mental health to legal problems among nonvictims.

Aim of the present study is to address these gaps in the literature.

#### First research question

The first research question was to what extent do people affected by violence, accidents, theft and/or serious threat (hereafter abbreviated as VATT) in the past 12 months and suffering from mental health, physical health, work, partner/ family, financial, administrative and legal problems, differ in the use of problem-related professional help compared to people not affected by these events in the past 12 months? More specifically, to what extent they 1.) currently receive problem-related professional help, 2.) do not need problem-related help, 3.) could use this help but do not use it, or 4.) cannot find or afford problem-related help [30] compared to nonvictims.

#### Second research question

The second research question was: to what extent do predictors of problem-related unmet needs differ between victims and nonvictims, such as experiences with other potentially traumatic or stressful life events, gender, age, education level, marital and employment status.

Before answering these questions, the extent to which victims compared to nonvictims more often suffered from the seven distinguished problems was examined.

## Materials and methods

### Procedures and participants

For the present study, data was extracted from three surveys of the longitudinal research project entitled Victims in Modern Society (VICTIMS) [6, 10, 31]. VICTIMS uses the Dutch Longitudinal Internet studies for the Social Sciences (LISS) panel for data collection. The set-up of this panel is funded by the Dutch Research Council (NWO) and is administered by CentERdata, The Netherlands [32]. It is based on a large traditional probability sample drawn from the Dutch population register by Statistics Netherlands (CBS). Respondents who do not have a computer and/or internet access are provided with the necessary equipment at home. Panel members receive an incentive of €15 per hour for their participation. For further information, see: https://www.dataarchive.lissdata.nl (in English).

The first wave of the VICTIMS study was conducted in March 2018 (T1) with reminders in April (N^invited^ = 7,292, response = 82.1%). The second wave was conducted in March 2019 (T2 follow-up) with reminders in April (N^invited^ = 6,298, response = 83.2%), and the third in March 2020 (T3) with reminders in April (N^invited^ = 6,568, response = 83.6%).

### Ethical approval and informed consent

In accordance with the current General Data Protection Regulation [33] participants gave explicit digital consent for the use of the collected data for scientific and policy relevant research. Our study and questionnaire was approved by an Internal Review Board of CentERdata, consisting of independent internal and external reviewers of CentERdata. These reviewers were not involved in the development of the research program. Since our research did not impose certain (experimental) behavior, our research did not need the approval of a Dutch Medical Ethical Testing committee according to the Dutch Law (see https://english.ccmo.nl-/investigators/legal-framework-for-medical-scientific-research/your-research-is-it-subject-tothe-wmo-or-not). Importantly, previous research using this panel revealed that the possible burden of participating in research on trauma was related to the perceived burden of other nontrauma studies such as on political values, but not with PTSD symptoms or other trauma-related variables [34].

### Measures

#### Violence, accidents, theft and serious threat

At T1, T2 and T3 respondents were administered a list of 21 potentially traumatic events (PTE’s) in the past 12 months, including violence, threats, accidents and theft-related events. This list with ‘yes’ or ‘no’ answer categories was based on previous research on PTE’s based on Criterion A1 events in DSM-IV, DSM-5 and events in the ICD-11. Participants were offered the opportunity to describe experienced PTE’s in the 12 months that were not listed. They were recoded in present or new categories of PTE (for details see [31]).

The victim group consisted of respondents victimized by:

1. physical violence, e.g. sexual violence/sexual abuse (not online), online sexual violence/sexual abuse, robbery, physical violence but not by own partner, and/or physical violence by own partner,
2. accidents, e.g. traffic accidents, disasters, fire, and/or medical errors,
3. theft, e.g. theft, robbery, burglary, and/or online theft, and/or
4. threat, e.g. serious threat without the use of physical violence (not online), online serious threat without use of physical violence.

The nonvictim group consisted of respondents not exposed to these events in the past 12 months.

Part of the victims and nonvictims were confronted with other PTEs or stress life-events in the past 12 months than VATT. Based on these data the dichotomous variable “exposure to other potentially traumatic and stressful life-events (PTE/SLE)” was constructed. Respondents who were confronted with one or more other potentially traumatic events (PTE) such as the unexpected loss of a significant other and/or stressful life-events (SLE) such as divorce were coded as “yes” and respondents not exposed to any event as “no”.

#### Current problems and use of problem-related professional help

At T1, T2 and T3 the brief screening Problems and Help Inventarisation-List (PHIL) [6, 35] was administered. This list examines problems in various domains such as mental health problems, physical problems, problems at work, problems in the family/with partner, financial problems, administrative problems, and legal problems (1 = yes, 2 = no).

In case respondents reported one or more problems, respondents were asked if they used professional help for their problem(s), such as from a general practitioner, psychiatrist, psychologist, lawyer, social worker, priest/ rabbi/ imam, financial expert, and victim assistance. These questions had the following exclusive answer categories for each reported problem separately: 1.) I do not need professional help for this problem, 2.) I am receiving professional help for this problem, 3.) I could use professional help for this problem, but I do not, 4.) I cannot find/ receive professional help for this problem, and 5.) I cannot afford professional help for this problem.

### Data analyses

#### Compile victim and nonvictim groups from the three surveys

To increase statistical power the three surveys were combined. In total, 7,212 respondents participated at T1, T2 and/or T3. Of this total study sample, 1,756 respondents reported in at least one survey that they were exposed to violence, accidents, theft and/or serious threat in the past 12 months, and they are labeled as victims. Because not all 7,212 respondents participated at all three surveys and several respondents were victimized in two or three periods, the victim group was composed as follows. First respondents were selected who were victimized by VATT in the 12 months before T1 (N = 739, 42.1% of 1,756). Next, respondents were selected who were exposed to VATT in the 12 months before T2 but not before T1, or did not participate at T1 (N = 485, 27.6% of 1,756). Finally, respondents were selected who were victimized by VATT in the 12 months before T3, but not before T1 and T2, or did not participate at T1 and/or T2 (N = 532, 30.3% of 1,756). In this way the victim group consisted of unique and independent records in the statistical analyses. Victims who reported in two or three surveys that they were exposed to VATT were used in the analyses only once: the VATT that was reported for the first time in the series of three measurements.

To compile a comparison group of nonvictims the following strategy was used. Of the total study sample, 5,456 respondents did not report that they were exposed to VATT in the past 12 months and they are labeled as nonvictims. Because the victim group was derived from three surveys, a nonvictims group was composed with a similar fraction of respondents at T1, T2 and T3 as the victim group. Because not all respondents participated in all three surveys, a random selection among all 5,456 nonvictims for T1, T2 and T3 respectively to match the fraction among victims was not possible. It resulted in a too low number of nonvictims at T3. This problem was solved by using a random selection of 2,100 nonvictims from the first survey, 1,400 nonvictims from the second survey (after excluding the selected 2,100 nonvictims from the first survey), and 1,500 from the third survey (after excluding the selected 2,100 and 1,400 nonvictims from the first and second survey; resulting in N^nonvictims^ = 5,000). In this way almost the same fraction across the three surveys as for the victim groups could be realized (T1: 42.0% versus 42.1%, T2: 28.0% versus 27.6% and T3: 30.0% versus 30.3%). For victims and nonvictims derived from the first survey (T1) data on demographics, problems and services use assessed at T1 were used in the analysis. For both groups derived from second survey (T2) data assessed at T2 were used and for both groups derived from the third survey data assessed at T3 were used. All analyses were conducted with IBM SPSS version 26. Differences between victims and nonvictims in demographics were assessed using chi-squared tests.

#### Analyses of differences in problems and use of professional help between victims and nonvictims with problems

To answer the research questions, a series of multivariate logistic regression analyses were conducted with victim status (1 = nonvictims, 2 = victims) as predictor. Sex, age, marital and employment status, education level, and exposure to other potentially traumatic and stressful life-events (PTE/SLE) were entered as control variables to prevent biased results as much as possible [36]. In the first series of analyses, the seven assessed problems and receiving professional help for these problems were the dependent variables.

To assess the extent to which victims with problems differed from nonvictims with similar problems in the use of professional help, similar logistic regression analyses were conducted for each problem separately. For this purpose, based on the four exclusive answer categories the following four dichotomous variables were created: 1.) Receiving professional help” (1 = no, 2 = yes); 2.) Do not need professional for problem” (1 = no, 2 = yes); 3.) Could use professional help, but do not use it” (1 = no, 2 = yes), and 4.) Can’t find or afford professional help” (1 = no, 2 = yes).

#### Analyses of differences in predictors unmet needs between victims and nonvictims

Unmet needs with respect to each assessed problem were defined as follows: respondents with problems who reported that they 3.) could use professional help, but did not use it, or 4.) could not find or afford professional help, were considered to have problem-related unmet needs.

First, multivariate logistic regression analyses were performed to examine the extent to which experiences with other PTE/SLE, sex, age, marital status, employment status and education level independently predicted problem-related unmet needs among victims (1 = no unmet needs, 2 = unmet needs). This analysis was repeated among nonvictims with similar problems.

Second, when at least one adjusted OR of a predictor was significant, the adjusted OR’s among both groups were compared by computing z scores (z = δ/SE(δ), where δ = AOR^victims^-AOR^nonvictims^ and SE(δ) = √ ((SE^log OR victims^)^2^ + (SE^log OR nonvictims^)^2^).

## Results

### Characteristics of victims and nonvictims

The characteristics of the victims and nonvictims group are presented in Table 1. Table 1 shows that the victims group compared to the nonvictims group consisted of significantly younger, less married, more unemployed and more highly educated respondents. They also were more often exposed to other potentially traumatic or stressful life-events. No difference in sex distribution was found.

**Table 1:** Characteristics of victims and nonvictims.

	Nonvictims (N = 5,000)	Victims (N = 1,756)			
	n (%)	n (%)	χ^2^	df	p
Sex					
• male	2,280 (45.6)	828 (47.2)	1.26	1	0.261
• female	2,720 (54.4)	928 (52.8)			
Age					
• 18–34 year	1,081 (21.6)	483 (27.5)	58.15	3	<0.001
• 35–49 year	1,055 (21.1)	404 (23.0)			
• 50–64 year	1,337 (26.7)	488 (27.8)			
• 65 year or older	1,527 (30.5)	381 (21.7)			
Married					
• no	2,394 (47.9)	1010 (57.5)	48.28	1	<0.001
• yes	2,606 (52.1)	746 (42.5)			
Employed					
• no	2,511 (50.2)	798 (45.4)	11.86	1	<0.001
• yes	2,489 (49.8)	958 (54.6)			
Education					
• prim. educ.	1,137 (22.7)	299 (17.0)	31.24	4	<0.001
• higher gen. sec.	326 (6.5)	116 (6.6)			
• inter. prof. educ.	1,180 (23.6)	401 (22.8)			
• high. prof. educ.	1,466 (29.3)	580 (33.0)			
• university	891 (17.8)	360 (20.5)			
Other PTE/SLE					
• no	3,436 (68.7)	917 (52.2)	154.37	1	<0.001
• yes	1,564 (31.3)	839 (47.8)			
VATT^1^					
• physical violence	n.a.	275 (15.7)	n.a.		
• accidents	n.a.	792 (43.4)			
• serious threat	n.a.	403 (22.9)			
• theft	n.a.	521 (29.7)			

VATT = exposed to physical violence, accidents, theft, and/or serious threat in past 12 months. PTE = potentially traumatic events in past 12 months. SLE = Stressful life-events in past 12 months. Education level: prim. educ. = primary education, preparatory intermediate vocational education; higher gen. sec. = higher general secondary/pre-university education; inter. prof. educ. = intermediate professional education; high. prof. educ = higher professional education. n.a. = not applicable.

^1^ Because some respondents are confronted with more than one type of event, the total percentage is higher than 100%.

### Prevalence of problems among victims and nonvictims

The prevalence of problems in various domains among both groups is shown in Table 2. When controlling for sex, age, marital status, employment status, education level, and exposure to other potentially traumatic and/or stressful life-events in the past 12 months, victims significantly more often had mental health, physical health, work, partner/family, financial, administrative, and legal problems.

**Table 2:** Problems among of victims and nonvictims.

	N^total^	Problems n (%)	aOR (95% CI)
Mental health problems			
• Nonvictims	5000	502 (10.0)	
• Victims	1756	339 (19.3)	1.85 (1.58–2.16)***
Physical problems			
• Nonvictims	5000	1709 (34.2)	
• Victims	1756	785 (44.7)	1.73 (1.54–1.95)***
Work problems			
• Nonvictims	2400	200 (8.3)	
• Victims	928	157 (16.9)	2.07 (1.64–2.60)***
Problems partner /family			
• Nonvictims	5000	356 (7.1)	
• Victims	1756	270 (15.4)	2.16 (1.81–2.57)***
Financial problems			
• Nonvictims	5000	330 (6.6)	
• Victims	1756	269 (15.3)	2.29 (1.91–2.73)***
Administrative problems			
• Nonvictims	5000	150 (3.0)	
• Victims	1756	148 (8.4)	2.62 (2.05–3.34)***
Legal problems			
• Nonvictims	5000	62 (1.2)	
• Victims	1756	79 (4.5)	3.26 (2.31–4.61)***

Victims = exposed to physical violence, accidents, theft, and/or serious threat in past 12 months. aOR = Odds Ratio adjusted for sex, age, marital status, employment status, education level, and exposure to other potentially traumatic and/or stressful life-events. 95% CI = 95% confidence interval adjusted Odd Ratio.

* p < 0.05

** p < 0.01

*** p < 0.001.

^1^ According to Dutch Law, in recent years the age at which people receive a state pension (AOW) is stepwise increased. To keep the maximum age of employed respondents similar across the three surveys, employed respondents were selected younger than 65 years old.

### Use of problem-related professional help among victims and nonvictims with problems

The prevalence of victims and nonvictims *with problems* that received professional help, did not need professional help, could use professional help but did not use it, and could not find or afford professional help for these problems are presented in Table 3. For example, of the 339 victims with mental health problems, 47.8% received professional help for these problems, 18.6% reported that they did not need professional help for these problems (no need), 20.1% reported that they could use professional help but did not do it (do not use), and 13.6% reported that they could not find or afford (cannot afford/find) professional help for these problems. Among the 502 nonvictims with mental health problems, the prevalence was 49.8%, 21.5%, 21.1% and 7.6%, respectively. Of all respondents with problems, a considerable minority did not use professional help although they considered it useful or did not use this help because they could not find or afford it such as for mental health problems (31%) and financial problems (37%).

**Table 3:** Use of professional help among victims and nonvictims with problems.

	Problem-related professional help							
	Receiving professional help		Do not need it		Could use it, but do not		Can’t find or afford it	
	n (%)	aOR (95% CI)	n (%)	aOR (95% CI)	n (%)	aOR (95% CI)	n (%)	aOR (95% CI)
Mental health problems								
• Nonvictims	250 (49.8)		108 (21.5)		106 (21.1)		38 (7.6)	
• Victims	162 (47.8)	0.93 (0.70–1.25)	63 (18.6)	0.84 (0.58–1.20)	68 (20.1)	0.94 (0.66–1.35)	46 (13.6)	1.84 (1.14–2.95)*
Physical problems								
• Nonvictims	1041 (60.9)		353 (20.7)		189 (11.1)		126 (7.4)	
• Victims	449 (57.2)	0.86 (0.72–1.03)	135 (17.2)	0.86 (0.68–1.07)	87 (11.1)	0.89 (0.67–1.18)	114 (14.5)	2.13 (1.61–2.83)***
Work problems^1^								
• Nonvictims	80 (40.0)		43 (21.5)		38 (19.0)		39 (19.5)	
• Victims	68 (43.3)	1.23 (0.78–1.92)	25 (15.9)	0.69 (0.39–1.22)	37 (23.6)	1.37 (0.80–2.33)	27 (17.2)	0.74 (0.42–1.32)
Problems partner /family								
• Nonvictims	87 (24.4)		153 (43.0)		82 (23.0)		34 (9.6)	
• Victims	72 (26.7)	1.14 (0.78–1.67)	94 (34.8)	0.71 (0.50–1.00)	51 (18.9)	0.77 (0.51–1.16)	53 (19.6)	2.28 (1.41–3.70)**
Financial problems								
• Nonvictims	45 (13.6)		182 (55.2)		50 (15.2)		53 (16.1)	
• Victims	48 (17.8)	1.15 (0.71–1.87)	100 (37.2)	0.55 (0.38–0.78)**	51 (19.0)	1.14 (0.72–1.81)	70 (26.0)	1.91 (1.24–2.93)**
Administrative problems								
• Nonvictims	45 (30.0)		45 (30.0)		33 (22.0)		27 (18.0)	
• Victims	26 (17.6)	0.54 (0.30–1.00)*	47 (31.8)	0.93 (0.54–1.60)	45 (30.4)	1.72 (0.97–3.02)	30 (20.3)	1.17 (0.63–2.20)
Legal problems								
• Nonvictims	36 (58.1)		11 (17.7)		6 (9.7)		9 (14.5)	
• Victims	32 (40.5)	0.44 (0.20–0.96)*	8 (10.1)	n.c	10 (12.7)	n.c.	29 (36.7)	4.86 (1.76–13.40)**

Victims = exposed to physical violence, accidents, theft, and serious threat in past 12 months. ref. = Reference category. aOR = Odds Ratio adjusted for sex, age, marital status, employment status, education level, and exposure to other potentially traumatic and/or stressful life-events. 95% CI = 95% confidence interval.

* p < 0.05

** p < 0.01

*** p < 0.001. n.c. = Not computed because of low cell counts (n^problem^ < 20).

^1^ Among employed respondents younger than 65 years old.

The results of the multivariate logistic regression analyses show that victims with problems compared to nonvictims with problems significantly more often could not find or afford professional help for their mental health, physical health, partner/ family, financial, and legal problems. In addition, victims with legal and administrative problems compared to nonvictims with these problems significantly less often received problem-related professional help. With respect to problems with partner/family and financial problems, victims significantly less often reported that they did not need professional help for their problems. No significant differences were found between both groups with respect to “could use professional help, but do not use it.

### Predictors of unmet needs among victims and nonvictims

In the present study, respondents were considered to have unmet needs when they could use professional help for their problems but did not use it, or could not find or afford help for their problems. The number of respondents among victims and nonvictims with problem-related unmet needs are presented in Table 3. For example, 114 victims and 144 nonvictims had unmet needs with respect to mental health problems. Due to low cell counts predictors of unmet needs related to legal problems could not be examined and compared.

In Table 4 the independent associations between predictors and unmet needs among victims and nonvictims are presented (adjusted OR’s). When adjusted OR’s of a predictor differed significantly between both groups and when at least one adjusted OR is significant, they are presented in ***bold italics*.** Below we focus on significant differences in predictors between both groups.

**Table 4:** Predictors of unmet needs among victims and nonvictims.

	Unmet needs for professional help related to the following problems					
	Mental health problems	Physical problems	Problems at work^1^	Problems with partner / family	Financial problems	Administrative problems
	aOR (95% CI)	aOR (95% CI)	aOR (95% CI)	aOR (95% CI)	aOR (95% CI)	aOR (95% CI)
Other PTE/SLE (no is reference)						
• Nonvictims	0.83 (0.54–1.27)	0.82 (0.63–1.06)	0.82 (0.43–1.55)	1.47 (0.92–2.35)	1.14 (0.68–1.91)	1.25 (0.61–2.58)
• Victims	1.42 (0.89–2.27)	0.95 (0.68–1.32)	0.84 (0.43–1.67)	1.28 (0.76–2.16)	1.23 (0.74–2.05)	1.10 (0.54–2.25)
Sex (man is reference)						
• Nonvictims	0.88 (0.58–1.34)	0.77 (0.60–1.00)*	***0*.*48 (0*.*27–0*.*89)*** *	0.60 (0.37–0.96)*	0.68 (0.42–1.11)	0.88 (0.44–1.78)
• Victims	0.70 (0.43–1.13)	0.75 (0.53–1.04)	***0*.*59 (0*.*29–1*.*17)***	0.68 (0.41–1.14)	0.58 (0.35–0.96)*	1.38 (0.66–2.85)
Age 35–49 (18–34 is reference)						
• Nonvictims	1.14 (0.64–2.02)	0.79 (0.52–1.22)	0.58 (0.27–1.27)	0.75 (0.36–1.56)	2.01 (0.95–4.29)	2.16 (0.65–7.13)
• Victims	0.89 (0.49–1.64)	0.92 (0.55–1.53)	1.74 (0.71–4.24)	1.07 (0.52–2.20)	1.24 (0.64–2.39)	1.48 (0.58–3.76)
Age 50–64 (18–34 is reference)						
• Nonvictims	1.70 (0.97–2.99)	0.52 (0.34–0.79)**	***0*.*79 (0*.*35–1*.*79)***	1.14 (0.55–2.35)	2.13 (1.02–4.44)*	1.09 (0.32–3.75)
• Victims	1.12 (0.61–2.04)	0.79 (0.48–1.30)	***2*.*50 (1*.*03–6*.*06)*** *	1.06 (0.53–2.11)	1.38 (0.70–2.74)	2.55 (0.95–6.81)
Age 65 and older (18–34 is reference)						
• Nonvictims	***2*.*75 (1*.*40–5*.*40)*** **	0.40 (0.26–0.61)***	n.a	1.71 (0.77–3.80)	2.10 (0.91–4.83)	1.93 (0.61–6.08)
• Victims	***1*.*26 (0*.*51–3*.*13)***	0.50 (0.29–0.87)*	n.a.	1.31 (0.54–3.20)	2.45 (1.00–6.04)	2.61 (0.84–8.15)
Married (no is reference)						
• Nonvictims	0.73 (0.46–1.14)	0.80 (0.62–1.05)	0.71 (0.38–1.34)	1.45 (0.89–2.35)	0.58 (0.35–0.98)*	0.52 (0.24–1.14)
• Victims	0.80 (0.47–1.37)	0.92 (0.64–1.31)	1.74 (0.87–3.52)	1.30 (0.75–2.24)	1.14 (0.64–2.02)	0.79 (0.39–1.62)
Employed (yes is reference)						
• Nonvictims	1.20 (0.76–1.89)	0.88 (0.65–1.21)	n.a.	1.27 (0.72–2.23)	1.33 (0.77–2.31)	0.86 (0.36–2.04)
• Victims	0.73 (0.44–1.19)	0.74 (0.51–1.07)	n.a.	1.00 (0.56–1.79)	0.72 (0.43–1.22)	0.94 (0.44–1.99)
Education higher gen. sec. (prim. educ. is reference)						
• Nonvictims	1.32 (0.55–3.16)	1.18 (0.70–1.99)	0.28 (0.04–1.85)	2.77 (0.97–7.86)	2.52 (0.95–6.65)	2.78 (0.63–12.18)
• Victims	1.03 (0.27–3.86)	0.49 (0.22–1.09)	0.26 (0.03–2.18)	0.78 (0.25–2.43)	0.59 (0.16–2.17)	0.33 (0.07–1.54)
Education inter. prof. educ. (prim. educ. is reference)						
• Nonvictims	1.06 (0.54–2.09)	1.06 (0.74–1.50)	0.40 (0.10–1.56)	1.28 (0.60–2.71)	1.39 (0.67–2.88)	2.30 (0.83–6.41)
• Victims	1.40 (0.66–3.00)	1.29 (0.80–2.10)	0.97 (0.22–4.36)	0.90 (0.38–2.10)	1.92 (0.96–3.85)	2.00 (0.72–5.55)
Education high. prof. educ. (prim. educ. is reference)						
• Nonvictims	1.51 (0.79–2.87)	0.85 (0.59–1.21)	0.37 (0.10–1.38)	0.81 (0.41–1.59)	1.30 (0.65–2.60)	2.24 (0.88–5.71)
• Victims	1.21 (0.57–2.54)	0.88 (0.54–1.43)	0.63 (0.14–2.74)	1.61 (0.75–3.47)	0.99 (0.47–2.08)	1.03 (0.37–2.82)
Education university (prim. educ. is reference)						
• Nonvictims	1.74 (0.88–3.44)	0.99 (0.65–1.51)	0.34 (0.09–1.32)	1.27 (0.60–2.67)	0.92 (0.41–2.07)	0.97 (0.27–3.50)
• Victims	2.00 (0.92–4.34)	0.81 (0.46–1.42)	1.06 (0.23–4.86)	0.78 (0.33–1.86)	0.94 (0.42–2.10)	2.26 (0.72–7.10)

Victims = exposed to physical violence, accidents, theft, and serious threat in past 12 months. aOR = Odds Ratio adjusted for all other variables in Table 4. 95% CI = 95% confidence interval adjusted Odd Ratio. Education level: prim. educ. = primary education, preparatory intermediate vocational education; higher gen. sec. = higher general secondary/pre-university education; inter. prof. educ. = intermediate professional education; high. prof. educ = higher professional education. n.a. = not applicable. Significantly different adjusted OR’s of a predictor with at least one significant adjusted OR are presented in bold italics.

* p < 0.05

** p < 0.01

*** p < 0.001.

^1^ Among employed respondents younger than 65 years old.

With respect to unmet needs for mental health problems, the results show that nonvictims of 65 years and older more often had unfulfilled needs than nonvictims of 18–34 years old (AOR = 2.75), but not among victims. The two adjusted OR’s differed significantly (z = 2.93, p = 0.003). The adjusted OR’s of all other assessed predictors were not significant.

Table 4 furthermore shows that both groups did not differ in predictors of unmet needs for physical problems. Older respondents (65 years and older) compared to young adults (18–34 years old) had significantly less often unmet needs for physical problems among both victims and nonvictims (AOR = 0.50 and AOR = 0.40 respectively) and the OR’s did not differ significantly. Although, for instance, sex was only significantly associated with less physical problem-related unmet needs among nonvictims (AOR = 0.48), the adjusted OR did not differ from the non-significant adjusted OR of the victims. Among victims 50–64 years old respondents had significantly more unmet needs with respect to problems at work than 18–34 years old respondents (AOR = 2.50), but not among nonvictims. The adjusted OR’s differed significantly (z = 3.08, p = 0.002).

With respect to partner/family-related, financial problems and administrative problems no significant differences between both groups in predictors of unmet needs were found.

## Discussion

The first aim of the present population-based study was to examine the use of problem-related professional help among victims of violence, accidents, theft and/or serious threat with problems, varying from mental health to legal problems, compared to the use of nonvictims with the similar problems. The second aim was to examine the extent to which predictors of problem-related unmet needs among victims, differed from predictors of problem-related unmet needs among nonvictims. As expected, the total victim group compared to the total nonvictim group more often experienced mental health, physical health, work, partner/family, financial, administrative, and legal problems [1–6].

### Difference in use of problem-related professional help

The main findings are that, depending on the type of problem, a variable minority (between about 7% to 37%) of both groups with problems could not find or afford problem-related professional help for physical problems, mental health problems, problems in the family/with partner, financial problems, and legal problems. Comparisons between both groups revealed that victims 1.5 to 2 times more often than nonvictims could not find or afford help for these problems. In addition, a substantial minority among the total study sample reported that they did not need professional help for their problems with partner/family (40%) and not for their financial problems (47%). However, victims significantly less often than nonvictims reported that they did not need professional help for problems with partner/family (35% versus 43%) and financial problems (37% versus 55%). victims also had significantly more often administrative problems than nonvictims, and victims with these problems less often used professional help for the problems (18% versus 30%). With respect to legal problems, victims significantly less often used professional help (41% versus 58%) and significantly more often reported that they could not find or afford professional help (37% versus 15%) for these problems. For problems at work, no differences in services use between both groups were found, and a similar percentage of victims and nonvictims with problems did not use problem-related professional help although they reported that they could use it.

These findings signal an important challenge for the various professionals, organizations, and institutions active in the field of victim assistance, to close these gaps. For instance, the outcome that about one out five to seven victims cannot find or afford problem-related professional help may serve as a strong indication that interventions should be developed (or re-designed) to solve these barriers of care for physical health, mental health, partner/ family, financial and legal problems. After all, services use among victims with problems should not be lower than among nonvictims with problems. Importantly, in all analyses differences between both groups in demographics and experiences with other potentially traumatic or stressful life-events in the past 12 months that may impact services use and thus may bias results were controlled for.

Receiving problem-related professional help for these problems varied strongly across the type of problems such as about 60% for physical problems, 49% for mental health problems and 16% for financial problems among the total study sample. The results with respect to mental health problems seem in line with previous research [12, 13] although the present study focused on current problems and current services use. With respect to these problems, victims with these problems used problem-related professional help as much as nonvictims. In the Netherlands, by law each citizen has a health insurance that provides access to a general practitioner and mental health services. Given this situation, the finding that victims more often report that they could not find or afford professional help related to mental health and physical problems is therefore counter-intuitive. Follow-up research is needed to gain insight in the reasons why they cannot find or afford this help. They also less often received help for administrative and legal problems. We are not aware of any population-based study in which services use for physical, work, partner/ family, financial, administrative, and legal problems among victims was compared with services use among nonvictims with similar problems to compare our findings with.

### Differences predictors problem-related unmet needs

Predictors of unmet needs were assessed among those with problems. Two general patterns can be observed. The first pattern is that several predictors were not significantly associated with unmet needs among both groups: experiences with other potentially traumatic or stressful life-events, employments status, and education levels. The second pattern that can be observed is that almost all significant predictors of unmet needs among victims did not differ significantly from predictors of unmet needs among nonvictims and vice versa. For instance, victims who experienced other potentially traumatic or stressful life-events did not more often have unmet needs related to mental health problems than victims without these experiences who also reported mental health problems and this pattern did not differ from nonvictims. However, this finding does not seem in line with previous research showing that multiple traumas increase the risk for PTSD that may increase the risk for unmet needs [37]. In contrast, older age (65+) was related to a higher prevalence of mental health-related unmet needs among nonvictims but not among victims. These findings suggest that there is no need that victims services should pay extra attention towards victims with other past-year traumatic or stressful events or older age to prevent mental health-related unmet needs as much as possible.

With respect to physical problem-related unmet needs, no significant differences between predictors were found; both older victims and older nonvictims (65+) had less unmet needs compared to young adults (18–34 years old) and the adjusted OR’s did not differ indicating that age had a similar effect among victims compared to nonvictims. A similar finding was observed for sex. Analyses among employed respondents revealed that victims of 50–64 years old were significantly more at risk for unmet needs related to problems at work than nonvictims, while female nonvictims were significantly more at risk than female victims.

It is difficult to compare these findings with the results of other studies because we are not aware of studies that systematically compared predictors of various problem-related unmet needs between victims and nonvictims. However, some findings seem in line with the study of Dezetter et al. [38] among patients with mental disorders according to the Perceived Need for Care Questionnaire (PNCQ) [39] to assess unmet needs. Like in our study, mental health related unmet needs were not related to sex, age, marital status and employment status.

### Strength and limitations

The use of a large population-based sample with high response rates, including both victims and nonvictims, may be considered a major strength of the present study. In addition, not only the prevalence of receiving problem-related help was assessed, but also the prevalence of ‘not needing help’, ‘not using help although it could be helpful’, and ‘cannot find or afford help’, in contrast to many other studies. Problem-related services use in the years before was not examined, indicating that recall bias does not play a role here [30]. In all analyses the possible effects of sex, age, marital status, employment status, education level, and exposure to other potentially traumatic and/or stressful life-events in the past 12 months were controlled for to prevent biased results as much as possible. It was outside the aim of the present study to examine the extent to which sex, age, marital status, employment status, education level, and exposure to other potentially traumatic and/or stressful life-events are associated with services use among victims with problems compared to nonvictims with similar problems. Assessed problems and use of problem-related professional help are based on self-reports. No clinical interviews were conducted. Unfortunately, given the numbers of respondents with problems it was not possible to examine services use among victims of violence, accidents, theft, and serious threat separately. It remains unclear to what extent these subgroups differ in services use compared to nonvictims. A broad range of problems was assessed but no further information is available about sub-categories of assessed problems or the duration of these problems. It is unknown from this study if victims face, for instance, other legal problems or other mental health problems than nonvictims. This study focused on potentially traumatic events in the past 12 months. Victims and nonvictims may have been victimized during earlier stages of their lives such as during their childhood and adolescence. Future studies should examine the role of these events in problem-related services utilization, including unmet needs and determinants, among victims and nonvictims.

We focused on relevant predictors such as experiences with other events and employment status, but were not able to examine other important predictors such as coping styles, co-morbidity, social cohesion and social solidarity in neighborhood settings [40], subjectively perceived barriers and facilitators with respect to assessed problems [41] that need to be addressed in future comparative studies. This study focused on adult victims and non-victims, and future studies are warranted to examine services use among children and adolescents that focus on age-related problems such as problems at school.

## Final conclusions

To the best of our knowledge, this is the first population-based study assessing the use of problem-related professional help among victims of violence, accidents, theft and serious treat with problems, compared to nonvictims with similar mental health problems, physical problems, problems at work, problems in the family/with partner, financial problems, administrative problems, and legal problems. In sum, this population-based study showed that victims more often suffer from problems than nonvictims, and if they suffer from problems there is a higher need for problem-related professional help than among nonvictims with the same problems. More specifically, individuals who are victimized by violence, accidents, theft and/or serious threat (VATT) in the past 12 months, more often suffer from all assessed problems than individuals not victimized by these events in this period. Dependent on the nature of the problem, victims with problems less often can find or afford problem-related professional help or less often receive problem-related professional help. Only with respect to problems at work, victims and nonvictims with these problems showed similar patterns in the use of professional help. In sum, there is a higher unmet need for different types of problems among victims than among nonvictims. Predictors of unmet needs among victims hardly differed from predictors among nonvictims.

When looking for possible ways to close this gap, our study can offer some directions. Examining the reason for not receiving problem-related professional help, the results show that it appears to be significantly more difficult for victims than for nonvictims to find or afford professional help. This indicates that the accessibility of various types of professional help for victims needs to and can be improved.

## References

[1] Eberhard-Gran M, Schei B, Eskild, A. Somatic symptoms and diseases are more common in women exposed to violence. J Gen Intern Med. 2008;22: 1668–1673. 10.1007/s11606-007-0389-8 PMC2219828 17922169

[2] O’Donnell ML, Creamer M, Elliott P, Atkin C. Health costs following motor vehicle accidents: the role of posttraumatic stress disorder. J Trauma Stress. 2005;18: 557–561. doi: 10.1002/jts.20064 16281254

[3] Loya RM. Rape as an economic crime: The impact of sexual violence on survivors’ employment and economic well-Being. J Interpers Violence. 2015;30: 2793–2813. doi: 10.1177/0886260514554291 25381269

[4] Mayou RA, Ehlers A, Bryant B. Posttraumatic stress disorder after motor vehicle accidents: 3-year follow-up of a prospective longitudinal study. Behav Res Ther. 2002;40(6): 665–675. doi: 10.1016/s0005-7967(01)00069-9 12051485

[5] Schatman ME, Thoman JL. Valid psychological injury claims: respecting the needs of survivors. Psychol Inj Law. 2015; 8: 311–322. 10.1007/s12207-015-9234-2

[6] Velden PG van der, Das M, Contino C, Knaap L van der. From health to financial problems: multi-problems among victims of partner and non-partner physical violence, and matched non-victims. J Interpers Violence. 2019. 10.1177/0886260519885915 PMC8581717 31686594

[7] Buitenhuis J, Jong PJ de, Jaspers JP, Groothoff JW. Relationship between posttraumatic stress disorder symptoms and the course of whiplash complaints. J Psychosom Res. 2006;61: 681–689. doi: 10.1016/j.jpsychores.2006.07.008 17084147

[8] Elbers NA, Hulst L, Cuijpers P, Akkermans AJ, Bruinvels DJ. Do compensation processes impair mental health? A meta-analysis. Injury. 2012;44: 674–683. doi: 10.1016/j.injury.2011.11.025 22244996

[9] Giummarra MJ, Ioannou L, Ponsford J, Cameron PA, Jennings PA, Gibson SJ, et al. Chronic pain following motor vehicle collision: A systematic review of outcomes associated with seeking or receiving compensation. Clin J Pain. 2016;32: 817–827. doi: 10.1097/AJP.0000000000000342 26889614

[10] Velden PG van der, Contino C, Marchand M, Das M, Schut H. Does pre-event lack of emotional support increase the risk of post-event PTSD, anxiety, depression symptoms and lack of support? A comparative population-based study among victims of threat and violence. J Anxiety Disord, 2020; 75. doi: 10.1016/j.janxdis.2020.102269 32795919

[11] Cho H., Kwon I., Shamrova D., & Seon J. (2019). Factors for formal help-seeking among female survivors of intimate violence. J Fam Violence. In press. 10.1007/s10896-019-00107-6

[12] McCart MR, Smith DW, Sawyer GK. Help seeking among victims of crime: a review of the empirical literature. J Trauma Stress. 2020;23(2): 198–206. 10.1002/jts.20509 PMC3803158 20336674

[13] Koenen K, Ratanatharathorn A, Ng L, McLaughlin K, Bromet E, Stein D, et al. Posttraumatic stress disorder in the World Mental Health Surveys. Psychol Med. 2017;47: 2260–2274. doi: 10.1017/S0033291717000708 28385165 PMC6034513

[14] Sripada RK, Pfeiffer PN, Valenstein M, Bohnert KM. Medical illness burden is associated with greater PTSD service utilization in a nationally representative survey. Gen Hosp Psychiatry. 2014; 36: 589–593. doi: 10.1016/j.genhosppsych.2014.09.007 25304762

[15] Lelaurain S, Graziani P, Lo Monaco G. Intimate partner violence and help-seeking. A Systematic Review and Social Psychological Tracks for Future Research. Eur Psychol. 2017;22: 263–281. 10.1027/1016-9040/a000304

[16] Robinson SR, Ravi K, Voth Schrag RJ. A Systematic Review of Barriers to Formal Help Seeking for Adult Survivors of IPV in the United States, 2005–2019. Trauma Violence Abuse. 2020. doi: 10.1177/1524838020916254 32266870

[17] Satyen L, Rogic AC, Supol M. Intimate partner violence and help-seeking behaviour: a systematic review of cross-cultural differences. J Immigr Minor Health. 2019; 21: 879–892. doi: 10.1007/s10903-018-0803-9 30105537

[18] Ullman SE. Mental Health Services Seeking in Sexual Assault Victims. Women Ther. 2007; 30: 61–84. 10.1300/J015v30n01_04

[19] Amstadter AB, McCauley JL, Ruggiero KJ, Resnick HS, Kilpatrick DG. Service utilization and help seeking in a national sample of female rape victims. Psychiatr Serv. 2008; 59: 1450–1457. doi: 10.1176/appi.ps.59.12.1450 19033173 PMC2735844

[20] Amstadter AB, Zinzow HM, McCauley JL, Strachan M, Ruggiero KJ, Resnick HS, et al. Prevalence and correlates of service utilization and help seeking in a national college sample of female rape victims. J Anxiety Disord. 2010; 24: 900–902. doi: 10.1016/j.janxdis.2010.06.014 20620018 PMC3687342

[21] US Bureau of Justice Statistics. National Crime Victimization Survey, Concatenated File, United States, 1992–2016. Inter-university Consortium for Political and Social Research, 2019-10-31. 10.3886/ICPSR36834.v3

[22] Belleghem G van, Deynse H van, Devos S, Huysmans E, Hubloue I. Health care utilization after hospitalization following a road traffic accident. J Disabil Rehabil. 2020; 42: 1599–1606. doi: 10.1080/09638288.2018.1531152 30616397

[23] Ruseckaite R, Gabbe B, Vogel AP, Collie A. Health care utilisation following hospitalisation for transport-related injury. Injury. 2012; 43: 1600–1605. doi: 10.1016/j.injury.2011.03.011 21466883

[24] Elbers NA, Cuijpers P, Akkermans AJ, Collie A, Ruseckaite R, Bruinvels DJ. Do claim factors predict health care utilization after transport accidents? Accid Anal Prev. 2013;53: 121–126. doi: 10.1016/j.aap.2013.01.007 23411157

[25] Scollay C. E., Berecki-Gisolf J., & Grant G.M. Trends in lawyer use in road traffic injury compensation claims. PLOSONE. 2020; 15, e0231025. doi: 10.1371/journal.pone.0231025 32251480 PMC7135282

[26] Elhai JD, North TC, Frueh BC. Health service use predictors among trauma survivors: A critical review. Psychol Services. 2005; 2: 3–19. 10.1037/1541-1559.2.1.3

[27] Lowe M, Willan VJ, Khan R, Brooks M, Robinson P, Graham-Kevan N, et al. Predictors of engagement with support services in a sample of UK victims of violence. Brit J Community Justice. 2016; 13: 21–34.

[28] Ford-Gilboe M, Varcoe C, Noh M, Wuest J, Hammerton J, Alhalal E, et al. Patterns and Predictors of Service Use Among Women Who Have Separated from an Abusive Partner. J Fam Violence. 2015; 30: 419–431. doi: 10.1007/s10896-015-9688-8 25960602 PMC4412644

[29] Lipsky S, Caetano R. Impact of intimate partner violence on unmet need for mental health care: Results from the NSDUH. Psychiatr Serv. 2007;58: 822–829. doi: 10.1176/ps.2007.58.6.822 17535943

[30] Roll JM, Kennedy J, Tran M, Howell D. Disparities in unmet need for mental health services in the United States, 1997–2010. Psychiatr Serv. 2013; 64: 80–82. doi: 10.1176/appi.ps.201200071 23280460

[31] Velden PG van der, Komproe I, Contino C, Bruijne M de, Kleber RJ, Das M, et al. Which groups affected by potentially traumatic events (PTEs) are most at risk for a lack of social support? A prospective population-based study on the 12-month prevalence of PTEs and risk factors for a lack of post-event social support. PLOSONE, 2020; 15. 10.1371/journal.pone.0232477 PMC7259781 32469880

[32] Scherpenzeel A, Das M. True Longitudinal and Probability-Based Internet Panels: Evidence from the Netherlands. In: Das M, Ester P, Kaczmirek L, editors. Social and Behavioral Research and the Internet: Advances in Applied Methods and Research Strategies. New York: Taylor & Francis; 2011. pp. 77–104.

[33] EU. Regulation (EU) 2016/679 of the European Parliament and of the council of 27 April 2016 on the protection of natural persons with regard to the processing of personal data and on the free movement of such data, and repealing Directive 95/46/EC (General Data Protection Regulation). 2016.

[34] Velden PG van der, Bosmans MWG, Scherpenzeel AC. The burden of research on trauma for respondents: A prospective and comparative study on respondents evaluations and predictors. PLOSONE. 2013; 8,10: e77266. doi: 10.1371/journal.pone.0077266 24204785 PMC3804544

[35] Velden, P.G. van der, Kleber, R.J. The Problems and Help Inventorisation-List (PHIL). Zaltbommel, The Netherlands: Institute for Psychotrauma/ Tilburg, The Netherlands: CentERdata. 1999/2018.

[36] Jankovic-Gavrilovic J, Schützwohl M, Fazal M, Priebe S. Who seeks treatment after a traumatic event and who does not? A review of findings on mental health service utilization. J Trauma Stress. 2005; 18: 595–605. doi: 10.1002/jts.20068 16382432

[37] Koenen KC, Harley R, Lyons MJ, Wolfe J, Simpson JC, Goldberg J, et al. A twin registry study of familial and individual risk factors for trauma exposure and posttraumatic stress disorder. J Nerv Ment Dis. 2002; 190: 209–218. doi: 10.1097/00005053-200204000-00001 11960081

[38] Dezetter A, Duhoux A, Menear M, Roberge P, Chartrand E, Fournier L. Reasons and Determinants for Perceiving Unmet Needs for Mental Health in Primary Care in Quebec. Can J Psychiatry. 2015; 60: 284–93. doi: 10.1177/070674371506000607 26175326 PMC4501586

[39] Meadows G, Harvey C, Fossey E, Burgess P. Assessing perceived need for mental health care in a community survey: development of the Perceived Need for Care Questionnaire (PNCQ). Soc Psychiatry Psychiatr Epidemiol. 2000; 35: 427–35. doi: 10.1007/s001270050260 11089671

[40] Fleury MJ, Ngui AN, Bamvita JM, Grenier G, Caron J. Predictors of Healthcare Service Utilization for Mental Health Reasons. Int J Environ Res Public Health. 2014; 11; 10559–10586. doi: 10.3390/ijerph111010559 25321874 PMC4210995

[41] Kantor V, Knefel M, Lueger-Schuster B. Perceived barriers and facilitators of mental health service utilization in adult trauma survivors: A systematic review. Clin Psychol Rev. 2017; 52: 52–68. doi: 10.1016/j.cpr.2016.12.001 28013081

